# Exercise Training Program Improves Subjective Sleep Quality and Physical Fitness in Severely Obese Bad Sleepers

**DOI:** 10.3390/ijerph192113732

**Published:** 2022-10-22

**Authors:** Pedro Delgado-Floody, Felipe Caamaño Navarrete, Luis Chirosa-Ríos, Cristian Martínez-Salazar, Claudia Andrea Vargas, Iris Paola Guzmán-Guzmán

**Affiliations:** 1 Department of Physical Education, Sport and Recreation, Universidad de La Frontera, Temuco 4811230, Chile; 2 Department Physical Education and Sports, Faculty of Sport Sciences, University of Granada, 18011 Granada, Spain; 3 Strength & Conditioning Laboratory, CTS-642 Research Group, Department Physical Education and Sports, Faculty of Sport Sciences, University of Granada, 18011 Granada, Spain; 4 Physical Education Career, Universidad Autónoma de Chile, Temuco 4780000, Chile; 5 Faculty of Chemical-Biological Sciences, Universidad Autónoma de Guerrero, Guerrero 39087, Mexico

**Keywords:** morbid obesity, exercise, sleep quality, quality of life

## Abstract

Background: Sleep quality is an important modulator of neuroendocrine function, as sleep problems are related to metabolic and endocrine alterations. Objective: The main objective was to determine the effects of an exercise training program on the sleep quality of severely obese patients with sleep problems. The secondary objective was to determine the relationship between fitness and anthropometric parameters with sleep quality scores. Methods: Thirty severely obese patients participated in 16 weeks of PA intervention (age: 39.30 ± 11.62 y, BMI: 42.75 ± 5.27 kg/m^2^). Subjective sleep quality, anthropometric parameters, and fitness (i.e., handgrip strength and cardiorespiratory fitness) were measured. Results: Two groups were defined as good sleepers (*n* = 15, 38.06 ± 12.26, men = 1) and bad sleepers (*n* = 15, 40.53 ± 11.23, men = 3). The good sleeper group reported improvement in cardiorespiratory fitness (61.33 ± 68.75 m vs. 635.33 ± 98.91 m, *p* = 0.003) and handgrip strength (29.63 ± 9.29 kg vs. 31.86 ± 7.17 kg, *p* = 0.049). The bad sleeper group improved their cardiorespiratory fitness (472.66 ± 99.7 m vs. 611.33 ± 148.75 m, *p* = 0.001). In terms of sleep quality dimensions, the bad sleeper group improved their subjective sleep quality (*p* < 0.001), sleep latency (*p* = 0.045), sleep duration (*p* = 0.031), and habitual sleep efficiency (*p* = 0.015). Comparing the changes in both groups (∆), there were differences in subjective sleep quality scores (∆ = 2.23 vs. ∆ = −3.90, *p* = 0.002), where 86.6% of the bad sleeper group improved sleep quality (*p* = 0.030). An increase in handgrip strength was correlated to improving sleep quality scores (r = −0.49, *p* = 0.050). Conclusions: Severely obese bad sleepers improved their subjective sleep quality, the components of sleep, and cardiorespiratory fitness through an exercise training program. Improvement in subjective sleep quality was linked to an increase in handgrip strength.

## 1. Introduction

Sleep quality comprises different subjective indices and global satisfaction parameters of sleep, where personal, social, and environmental factors may affect sleep health [1,2]. Sleep quality plays a relevant role in long-term health across the entire lifespan [3]. In addition, evidence suggests that meeting sleep duration recommendations is related to health benefits [4]. Conversely, a previous study has shown that poor sleep quality was associated with health risk behaviors [5] and adverse health outcomes [6], including cardiovascular disease [7], obesity [8], and all causes of mortality [9,10]. Similarly, poor sleep quality has been related to less well-being [11] and an increase in public health costs [12]. In this sense, a previous study conducted in Australia reported that the cost of sleep disorders was around USD 35.4 billion in 2019–2020 [13]; hence, today, sleep disturbances are considered a public health problem [14].

Poor sleep quality has been indicated as a risk factor for obesity [15,16]. In this sense, previous studies have documented associations between the types of obesity and sleep problems [17]. In addition, bad sleep quality may predict obesity and other anthropometric parameters, such as body fat mass [18]. Previous evidence showed that sleep quality problems were related to more leptin secretions and visceral adipose tissue [19]. Another previous study suggested that inadequate sleep was associated with obesity and being overweight; however, a causal link could not be determined [20]. Likewise, it has been indicated that sleep quality subtype alterations could be a risk of obesity; this investigation reported that poor sleepers had the highest body mass index, compared with the other groups [21]. Additionally, a study indicated that poorer sleep quality was linked with greater food intake and bad quality diet [22]. Therefore, it is necessary to develop strategies to improve sleep quality.

In relation to the above, a systematic review reported that an exercise training program had promising results on sleep quality [23]. In addition, a previous study showed that 8 weeks of regular aerobic exercise increases different components of sleep quality [24]. Likewise, another study reported that sleep quality significantly improved after an 8-week intervention through exercise-induced weight loss [25]; however, another study indicated that the effect sizes of intervention (using high intensity) on sleep quality were affected by the type, duration, and frequency of the intervention [26]. Moreover, evidence showed that exercise training programs increased physical fitness (i.e., cardiorespiratory fitness and muscular strength) [27], had positive effects on weight loss and body composition changes [28], and improved subjective sleep quality [29] in obese patients. However, little is known about the benefits of an exercise training program on the sleep quality of bad sleepers with severe/morbid obesity; therefore, the effects of subjective sleep quality dimensions must deeply be investigated in this population. Hence, the main objective of the present study was to determine the effects of an exercise training program on the subjective sleep quality and physical status (i.e., weight status and fitness) of severely obese patients with sleep problems. A secondary objective was to determine the relationship between anthropometric parameters and physical fitness with sleep quality scores.

## 2. Materials and Methods

### 2.1. Study Design

This was a quasi-experimental study developed in patients with severe/morbid obesity that evaluated the effects of 16 weeks of an exercise training program (2 times per week, total; 32 sessions). The patients were invited for participating through a public invitation and directly to the Morbidly Obesity Association of Temuco, City, Chile (OBEMOB). After providing all the information and feedback about risks/benefits, all the participants signed informed consent. The study was carried out following the Declaration of Helsinki (2013) and was approved by the Ethical Committee of the Universidad de La Frontera, Temuco, Chile (DI21-0030 Project, ACTA Nº 080_21).

### 2.2. Patients and Recruitment

The inclusion criteria were as follows: (i) age 18–60 years, (ii) medical authorization for physical exercise, and (iii) a body mass index (BMI) equal to or greater than 40.0 or 35.0–39.9 with obesity-related health conditions (hypertension, diabetes type 2, insulin resistance, etc.). The exclusion criteria were (i) physical limitations to performing the physical tests (e.g., the restrictive injuries of the musculoskeletal system), (ii) exercise-related dyspnea or respiratory alterations, and (iii) chronic heart disease with any degree of worsening in the last month. After the enrollment stage, forty-four (*n* = 44) participants were assessed for eligibility, and nine (*n* = 9) were not included according to the inclusion criteria. For the first evaluation of sleep quality (*n* = 35), the groups (pretest) were designated based on the global Pittsburgh Sleep Quality Index (PSQI) report, with a score of <5 denoting high sleep quality (i.e., good sleepers, *n* = 18) and a score of ≥ 5 denoting poor sleep quality (i.e., bad sleepers, *n* = 17). After the loss of follow-up participants (good sleepers, *n* = 3 and bad sleepers, *n* = 2) for data analysis, thirty (*n* = 30) participants were included in the final sample size (age: 39.3 ± 11.62 y, BMI: 42.75 ± 5.27 kg/m^2^). The groups were as follows: good sleepers (*n* = 15, 38.06 ± 12.26 and, men = 1) and bad sleepers (*n* = 15, 40.53 ± 11.23 and, men = 3).

### 2.3. Measurements

#### 2.3.1. Sleep Quality Measurements

Sleep quality was assessed using the PSQI [30]. The PSQI is a self-reported questionnaire that includes seven component scores: (i) subjective sleep quality, (ii) sleep latency, (iii) sleep duration, (iv)habitual sleep efficiency, (v) sleep disturbances, (vi) use of sleeping medication, and (vii) daytime dysfunction. In the PSQI, subjects rated their perceived sleep quality as very good, fairly good, fairly bad, or very bad. These subjective scales are weighted to obtain a global PSQI score that differentiates between good and poor sleep quality. Their sum builds the global PSQI report, which provides an ”inverse score”, where a score < 5 denotes high sleep quality (i.e., good sleepers) and a score ≥ 5 denotes poor sleep quality (i.e., bad sleepers). This scale has been used in previous studies [18] that examined effects in bariatric patients [31]. Conditions associated with PSQI included the use of sleep medications, difficulties in daily living and enthusiasm, and low sleep efficiency. Sleep quality was evaluated 48 h before starting the intervention and 48 h after the last session.

#### 2.3.2. Anthropometric Parameters

Body mass (kg) was measured using a digital bioimpedance scale (TANITA^TM^, model 331, Tokyo, Japan). Height (m) was measured with a SECA^TM^ stadiometer (model 214, Hamburg, Germany), with subjects in light clothing and without shoes. The BMI was calculated as the body weight divided by the square of the height (kg/m^2^). The BMI was determined to estimate the degree of obesity (kg/m^2^) using the standard criteria for obesity and morbid obesity classification [32,33]. The anthropometric parameters were measured after fasting (6 ± 2 h) 48 h before starting the intervention and 48 h after the last session.

#### 2.3.3. Physical Fitness

Before starting the intervention, the physical condition of the participants was measured through endurance and muscle strength testing. First, a six-minute walking test (6Mwt) was used to determine cardiorespiratory fitness (CRF). During the test, the participants were assisted with instructions from an exercise physiologist [34]. Handgrip strength (HGS) was assessed using a digital dynamometer (Baseline^TM^ Hydraulic Hand Dynamometers, USA), which has been used in previous studies [35]. Two attempts were made, measuring each hand, and the best result from each was selected. The mean value regarding both the best left- and right-hand records was taken as the total score [35]. Physical fitness was evaluated 48 h before starting the intervention and 48 h after the last session.

#### 2.3.4. Exercise Training Intervention

An exercise training program was carried out in the training center and laboratory (UFRO, Temuco, Chile) in groups, and it was applied 2 days per week (Tuesday and Thursday) with two sections. First, the resistance training (RT) section included three out of four exercises targeting the following different muscle groups: (i) forearm, (ii) knee flexors and extensors, (iii) trunk, (iv) chest, (v) shoulder elevators, (vi) horizontal shoulder flexors, (vii) extensors, and (viii) plantar flexors. These exercises were performed in 3 sets of as many repetitions (continuous concentric/eccentric voluntary contraction) as possible in 60 s (intensity; 40–60% 1RM), followed by 60 to 120 s of passive recovery, as previously reported [36]. Before starting the session, the participants were evaluated to estimate the intensity (%) in the different RT exercises, and the maximum dynamic muscular strength (1RM) was indirectly estimated through the Brzycki formula [37], with fewer than 12 maximum repetitions.

Second, a high-intensity interval training (HIIT) section consisted of 60 s of maximum intensity exercise using a magnetic resistance static bicycle (Oxford^TM^ Fitness, model BE-2701, Santiago, Chile), followed by 60–120 s of passive recovery over the bicycle off. This was repeated four to seven times [18]. The exercise intensity was measured on the Borg scale (1 to 10 of perceived exertion), and the participants worked at a level between 6 and 9 points. All the sessions started with a 10 min warm-up period with continuous walking and joint mobility and flexibility exercises, followed by 5–10 min of cool down and stretching to prevent injuries. Each exercise training session had a time duration of 60 min/session, accumulating 120 min/week.

### 2.4. Data Analysis

This procedure was performed using the SPSS statistical software, version 23.0 (SPSS™ Inc., Chicago, IL, USA). The absolute frequencies were determined for the qualitative variables. Comparisons between the groups were evaluated using a Student’s *t*-test. To determine the changes from pre- to post-test, a repeated-measure two-way ANOVA (group × 2 times) was applied, and the delta (Δ) changes were calculated. Cohen’s d effect size was obtained with threshold values at 0.20, 0.60, 1.2, and 2.0 for “small”, “moderate”, “large”, and “very large” effect sizes, respectively [38]. In order to determine the linear correlation between sleep quality score and anthropometric, metabolic, and fitness parameters, Pearson’s correlation coefficients were calculated. The changes (%) in the sleep quality category (better or worse) between the pre-test and the post-test were evaluated using the McNemar test. Values of *p ≤* 0.05 were considered statistically significant.

## 3. Results

Table 1 shows the baseline characteristics of the sample study according to sex (female/male 26/4), age (39.3 ± 11.62 y), physical status (BMI; 42.75 ± 5.27 kg/m^2^), and subjective sleep quality (5.96 ± 3.71 score).

Table 2 reports the changes in the study variables after the intervention. Based on sleep quality, the groups were as follows: good sleepers (*n* = 15, 38.06 ± 12.26 and, men = 1) and bad sleepers (*n* = 15, 40.53 ± 11.23 and, men = 3). The good sleeper group reported improvement in cardiorespiratory fitness (561.33 ± 68.75 m vs. 635.33 ± 98.91 m, *p* = 0.003, ES: 0.87) and handgrip strength (29.63 ± 9.29 kg vs. 31.86 ± 7.17 kg, *p* = 0.049, ES: 0.27). The bad sleeper group reported significant changes in BMI (45.32 ± 7.51 kg/m^2^ vs. 44.33 ± 7.28 kg/m^2^, *p* = 0.005, ES: 0.13) and an improvement in cardiorespiratory fitness (472.66 ± 99.7 m vs. 611.33 ± 148.75 m, *p* = 0.001, ES: 1.10). The bad sleeper group improved their subjective sleep quality (*p* < 0.001, ES: 1.27), sleep latency (*p* = 0.045, ES: 0.83), sleep duration (*p* = 0.031, ES:0.84), and habitual sleep efficiency (*p* = 0.015, ES: 0.99).

**Figure 1:**
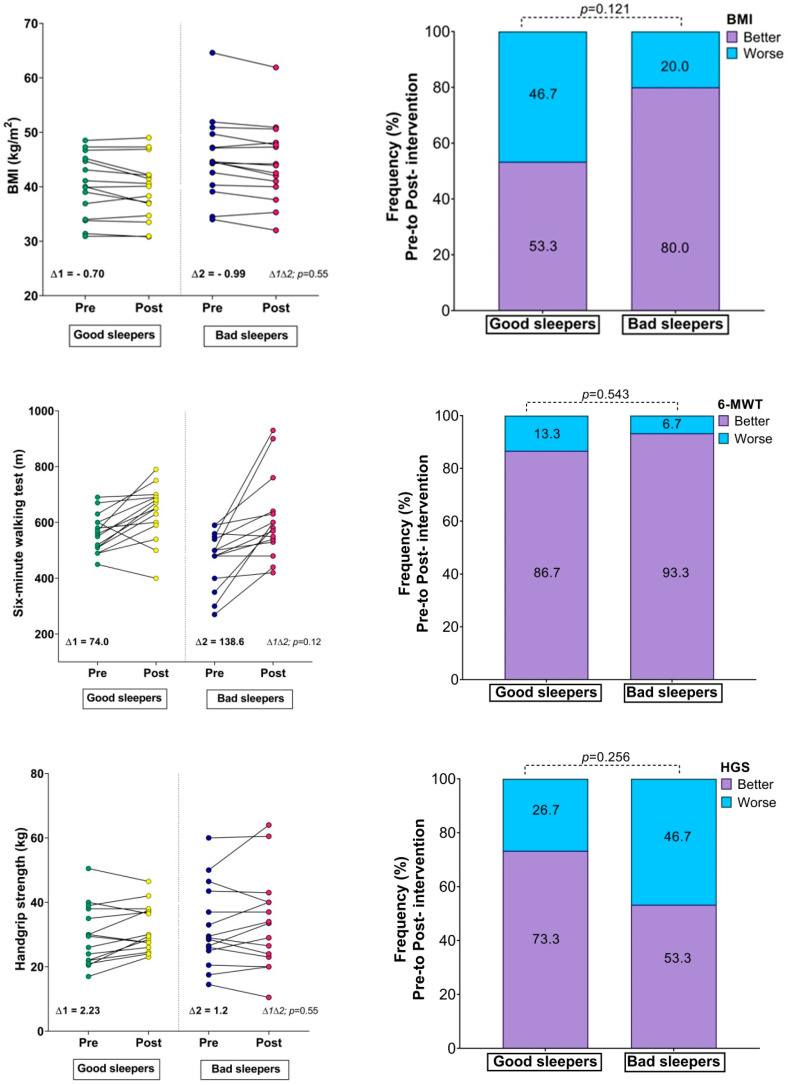

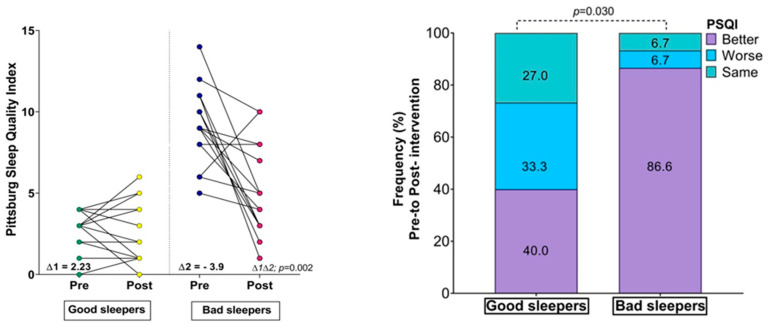
Changes in body mass index, physical fitness, and Sleep Quality Index; (∆) denotes delta changes from pre- to post-intervention. BMI = body mass index. *p*-value represents group × time interaction and McNemar test.

**Figure 2:**
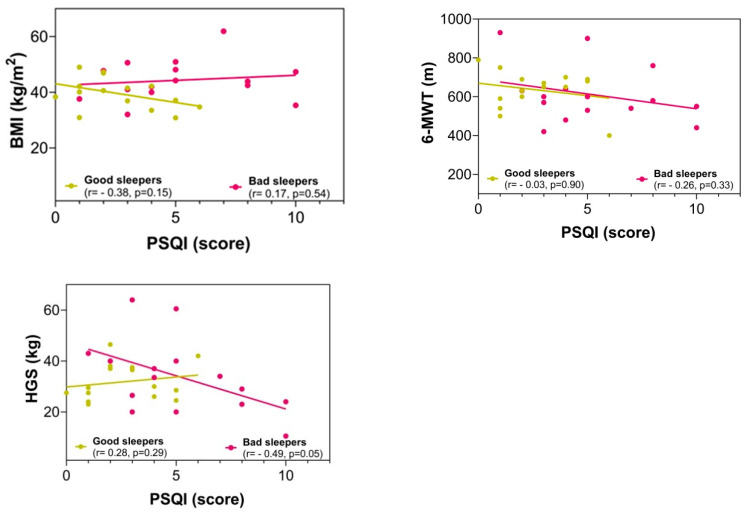
Correlation between the sleep quality score with anthropometric and fitness variables in post-intervention. BMI = body mass index; 6-MWT = six-minute walking test; HGS = handgrip strength; PSQI = Pittsburgh Sleep Quality Index.

There were no significant differences between the groups in the fitness comparisons (Figure 1). Comparing both groups with regard to the delta (∆) changes from pre- to post-test, there were significant differences related to time × group interaction in subjective sleep quality scores (∆ = 2.23 vs. ∆ = −3.90, *p* = 0.002), where 86.6% of the bad sleeper group improved sleep quality (*p* = 0.030) (Figure 1). Improved handgrip strength was correlated with an improvement in the sleep quality score (r = −0.49, *p* = 0.05) (Figure 2).

## 4. Discussion

The main objective of the present study was to determine the effects of exercise training programs on the sleep quality of severely obese patients with sleep problems. A secondary objective was to determine the relationship between fitness and anthropometric parameters with sleep quality scores. The main results were as follows: (i) sleep quality improved in the severely obese bad sleepers; (ii) the different components of sleep quality (i.e., subjective sleep quality, sleep latency, sleep duration, and habitual sleep efficiency) improved; (iii) an increase in handgrip strength was related to improvement in the sleep quality of bad sleepers; and (iv) the exercise training program improved the physical status (i.e., BMI and cardiorespiratory fitness).

The sleep quality of the bad sleeper group improved in comparison with that of the control group (good sleepers). This is an interesting result due to the evidence that suggests good sleep is related to different health benefits [4]. In this sense, an exercise study on obese young subjects indicated that 12 weeks of supervised exercise training (160–180 min of moderate to intense training per week, including 30 min of aerobic exercise plus 20 min of resistance training) positively impacted sleep quality and sleep duration [39]. In addition, previous evidence derived from cluster analysis showed that poorer sleepers had lower PA levels than their counterparts [40]. In this context, six months of an exercise training program (cycle ergometer and treadmill exercises at 50% of VO_2_ peak) improved sleep quality in sedentary and overweight/obese women [41]. Likewise, it was reported that a 12-week exercise training program (150 min/week of moderate-to-vigorous PA followed by resistance training) improved sleep quality and reduced the screening apnea–hypopnea index in overweight/obese adults [42]. Conversely, another study of a 12-week exercise program intervention (a progressive walking program plus an optional resistance training program) reported that good and poor sleepers did not improve sleep quality [43].

The different components of sleep quality (i.e., subjective sleep quality, sleep latency, sleep duration, and habitual sleep efficiency) improved with the intervention, as 86.6% of the participants improved sleep quality with the intervention. These results are relevant since evidence has shown that the different sleep components present favorable associations with health outcomes such as mental health, cognitive function, emotional well-being, and physical health [4]; furthermore, it has been suggested that improving sleep by exercise training could also result in many benefits, including variations in sleep qualitative parameters [44]. Similarly, a study of 6 weeks of the HIIT program indicated that the participants had a greater PSQI global score than the control group (21.3% increase), which means moving from “poor sleep quality” to “good sleep quality” [45]. Likewise, a previous study reported that a 12-week HIIT program positively impacted the PSQI score, sleep latency, sleep disturbances, and sleep quality [46]. Another intervention study showed that exercise training induced an improvement in subjective sleep quality in sedentary middle-aged adults; moreover, the HIIT group reported an improvement in the objective sleep quality parameters (total sleep time, sleep efficiency, and wake after sleep onset) after 12 weeks of exercise intervention [47]. A longitudinal study showed that having a healthy lifestyle (i.e., being physically active) could be a good strategy to improve the sleep quality of obese participants [48].

We found that an increase in handgrip strength was related to improved sleep quality in bad sleepers. Like our results, previous evidence has demonstrated that lower HSG was negatively related to PSQI score [49]. Likewise, another study reported a positive relationship between muscle mass and sleep quality [50]. In the context of morbid and severe obesity, a previous study reported that poor sleep quality had a positive association with several negative outcomes, including body fat percentage, glucose alteration, and poorer HSG [51]. In addition, in obese subjects, improvements in sleep quality have been reported after participating in a strength program [52]. Another study reported that physical fitness (i.e., HGS) predicted sleep quality problems [53].

Finally, the exercise training program improved the physical status (i.e., BMI and cardiorespiratory fitness). These results are in line with the evidence indicating reduced risk factors of mortality [54]. Similarly, a previous study found that a 12-week exercise intervention (3 × 90 min exercise session per week) had positive effects on cardiorespiratory fitness, muscular endurance, and body composition in overweight and obese adults [55]. In addition, another study showed that an exercise intervention using two modalities (continuous and interval training) induced weight loss, reduced BMI, and increased cardiorespiratory fitness in obese subjects [56]. The results could be explained by the fact that exercise training increases resting energy expenditure, lipid oxidation, and mitochondrial capacity [57].

## 5. Limitations

A limitation of the study was that sleep quality was measured using a questionnaire in a subjective way. Additionally, steps per day, sedentary behaviors, and eating habits were not controlled during the intervention; however, each week, the participants were reminded not to change their baseline patterns. By contrast, a strength of this study was that we included the different components of subjective sleep quality in bad sleepers, which are relevant as indicators to improve the quality of life of the participants.

## 6. Conclusions

In conclusion, severely obese bad sleepers improved their subjective sleep quality, different sleep components, and cardiorespiratory fitness through an exercise training program. The improvement in subjective sleep quality was linked to an increase in handgrip strength. Therefore, obese patients with poor sleep quality can incorporate exercise to improve sleep quality, as a treatment or as a complement to their usual treatment.

## Figures and Tables

**Table 1:** Shows the characteristics of sample study.

*Participant Characteristics*	Sample *n* = 30
Age	39.30 ± 11.62
Sex: Female/Male	26/4
***Anthropometric***	
Size (m)	1.59 ± 0.08
Body mass (kg)	108.82 ± 21.48
BMI (kg/m^2^)	42.75 ± 5.27
Obesity category	
Moderate–Severe (30–39.9 kg/m^2^), *n* (%)	10 (33.33)
Morbid obesity (≥40 kg/m^2^), *n* (%)	20 (66.66)
***Physical fitness***	
6-MWT (m)	517 ± 95.48
HGS (kg)	31.05 ± 11.03
***PSQI test***	
PSQI score	5.96 ± 3.71
Sleeper group	
Good sleepers *n* (%)	15 (50.0)
Bad sleepers *n* (%)	15 (50.0)

The data shown represent the mean ± standard deviation. BMI = body mass index; PSQI = Pittsburgh Sleep Index Quality; 6-MWT = six-minute walking test; HGS = handgrip strength.

**Table 2:** Changes in study variables after physical activity intervention.

Groups	Good Sleepers (Control) *n* = 15				Bad sleepers (Cases) *n* = 15			
*Participant Characteristics*	Pre-Intervention	Post-Intervention	*p*-Value	Cohen d	Pre-Intervention	Post-Intervention	*p*-Value	Cohen d
Age	40.00 ± 14.26	-	-		40.50 ± 11.23	-	0.570	
Sex Female/Male	14/1	-			12/3	-	0.283	
***Anthropometric***								
Size (m)	1.58 ± 0.068	-			1.60 ± 0.106	-	0.546	
Body mass (kg)	102.30 ± 20.0	99.50 ± 19.0	0.068	0.09	116.37 ± 21.31 *	113.91 ± 21.06 ^¥^	**0.008**	0.12
BMI (kg/m^2^)	40.20 ± 6.0	39.50 ± 6.0	0.080	0.12	45.32 ± 7.51 *	44.33 ± 7.28 ^¥^	**0.005**	0.13
Obesity category							0.121	
35–39.9 kg/m^2^	7 (46.7)	-			3 (20.0)	-		
≥40 kg/m^2^	8 (53.3)	-			12 (80.0)	-		
***Physical fitness***								
6MWT (m)	561.33 ± 68.75	635.33 ± 98.91	**0.003**	0.87	472.66 ± 99.74	611.33 ± 148.75	**0.001**	1.10
HGS (kg)	29.63 ± 9.29	31.86 ± 7.17	**0.049**	0.27	32.46 ± 12.71	33.66 ± 14.66	0.404	0.09
***PSQI test***								
PSQI score	2.80 ± 1.20	2.66 ± 1.79	0.757	0.09	9.13 ± 2.38 *	5.2 ± 2.80 ^¥^	**0.001**	1.51
Sleep Quality Dimensions								
Subjective sleep quality (score)	0.13 ± 0.35	0.26 ± 0.59	0.334	0.27	0.93 ± 0.70	0.20 ± 0.41	**<0.001**	1.27
Sleep latency(score)	0.60 ± 0.50	0.66 ± 0.61	0.581	0.11	1.80 ± 0.77	1.2 ± 0.67	**0.045**	0.83
Sleep duration(score)	0.66 ± 0.61	0.4 ± 0.50	0.103	0.47	1.66 ± 1.04	0.8 ± 1.01	**0.031**	0.84
Habitual sleep efficiency (score)	0.33 ± 0.48	0.53 ± 0.63	0.271	0.40	2.26 ± 1.09	1.13 ± 1.18	**0.015**	0.99
Sleep disturbances(score)	0.66 ± 0.48	0.6 ± 0.50	0.334	0.12	1.2 ± 0.41	1.0 ± 0	0.082	0.70
Sleeping medication (score)	0 ± 0	0 ± 0	-		0.6 ± 0.91	0.33 ± 0.72	0.217	0.33
Daytime dysfunction (score)	0.4 ± 0.73	0.2 ± 0.41	0.082	0.34	0.66 ± 0.61	0.53 ± 0.51	0.498	0.23

The data shown represent the mean ± standard deviation. BMI = body mass index; PSQI = Pittsburgh Sleep Index Quality; 6-MWT = six-minute walking test; HGS = handgrip strength. The effect size (ES) was calculated using Cohen’s d; * denotes significant differences (*p* < 0.05) between groups at baseline; ^¥^ denotes significant differences in group × time interaction (*p* < 0.05). Significant differences are in bold.

## Data Availability

Not applicable.
